# Myotonic dystrophy type 1 presenting with stroke-like episodes: a case report

**DOI:** 10.1186/1756-0500-6-243

**Published:** 2013-06-26

**Authors:** Jens D Rollnik, Ute Heinz, Olaf Lenz

**Affiliations:** 1 BDH-Clinic Hessisch Oldendorf, Teaching Hospital of Medical School Hannover, Institute of Neurorehabilitational Research (InFo), Greitstr. 18-28, Hessisch Oldendorf 31840, Germany

## Abstract

**Background:**

It is well known that myotonic dystrophy type 1 (DM1) - Curschmann-Steinert disease - is associated with white matter lesions in the brain. Further, DM1 patients may suffer from cardiac involvement and cardioembolic strokes. We report on the unique case of an adult-onset DM1 without cardiac or vascular abnormalities presenting with stroke-like episodes.

**Case presentation:**

A 40 y old white female was admitted twice to our stroke unit with apoplectic dizziness, nausea, headaches, and numbness in the right arm. She was suffering from type 2 diabetes, cataract, and endometriosis. Magnetic resonance imaging (MRI) revealed confluent white matter lesions in all cerebral lobes. There was no hyperintensity on diffusion-weighted imaging (DWI) and no gadolinium enhancement. Cerebrospinal fluid was normal. Surprisingly, myotonic discharges were detected in electromyography (EMG). Genetic testing revealed 200 ± 10 CTG repeats in the dystrophia myotonica protein kinase (DMPK) gene on chromosome 19 and DM1 was diagnosed.

**Conclusions:**

DM1 may be the cause of cerebral white matter lesions. This is the first case of DM1 presenting with stroke-like episodes.

## Background

Myotonic dystrophy type 1 (DM1, Curschmann-Steinert disease) is considered to be the most common adult-onset muscular dystrophy [1]. In a European population, DM1 has an estimated prevalence of about 1:10.000 [1]. DM1 is an autosomal-dominantly inherited disease caused by a trinucleotide (cytosine-thymine-guanine = CTG) expansion mutation in the dystrophia myotonica protein kinase (DMPK) gene on chromosome 19 [1]. In DM1, the length of the mutation correlates with disease severity and age of onset [1]. Phenotype includes myotonia, limb muscle weakness, muscle atrophy, facial weakness, ptosis, and multi-organ involvement including cataracts, insulin resistance, elevated liver enzyme levels, male hypogonadism and cardiac conduction defects [1]. DM1 may also present with peripheral nervous manifestation (polyneuropathy) [2].

It is well known that DM1 is associated with cognitive impairment, caused by cerebral white matter lesions and brain atrophy [3-6].

There are various cardiac manifestations in DM1 associated with high cardiac morbidity and mortality: atrioventricular block, ventricular premature contractions, atrial fibrillation/flutter, right/left bundle branch block, non-sustained ventricular tachycardia, sudden cardiac death, mitral valve prolaps, and left ventricular systolic dysfunction [7,8]. Due to atrial fibrillation/flutter DM1 patients may suffer cardioembolic strokes but the incidence of cerebral infarctions is quite low (1.5% in a 17 y observation period) [9].

Myotonic dystrophy type 2 (proximal myotonic myopathy = PROMM) may be associated with stroke-like episodes [10]. However, among DM1 patients no stroke-like symptoms have been reported, yet.

## Case presentation

We report on a 40 y old white female admitted twice to our stroke unit (June and October 2012) because of apoplectic dizziness, nausea, headaches, and numbness in the right arm. In addition, the patient was suffering from type 2 diabetes, cataract, and endometriosis.

In the clinical neurological examination, there was a facial but no limb muscle weakness or atrophy. Ankle jerks were weak. Symptoms significantly improved after a couple of days.

**Figure 1:**
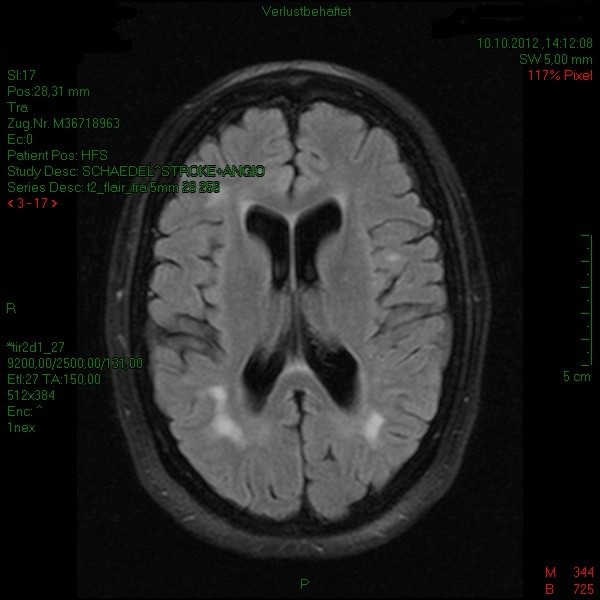
**T2-weighted cerebral MRI scan.** There are confluent white matter lesions in all cerebral lobes and signs of brain atrophy.

**Figure 2:**
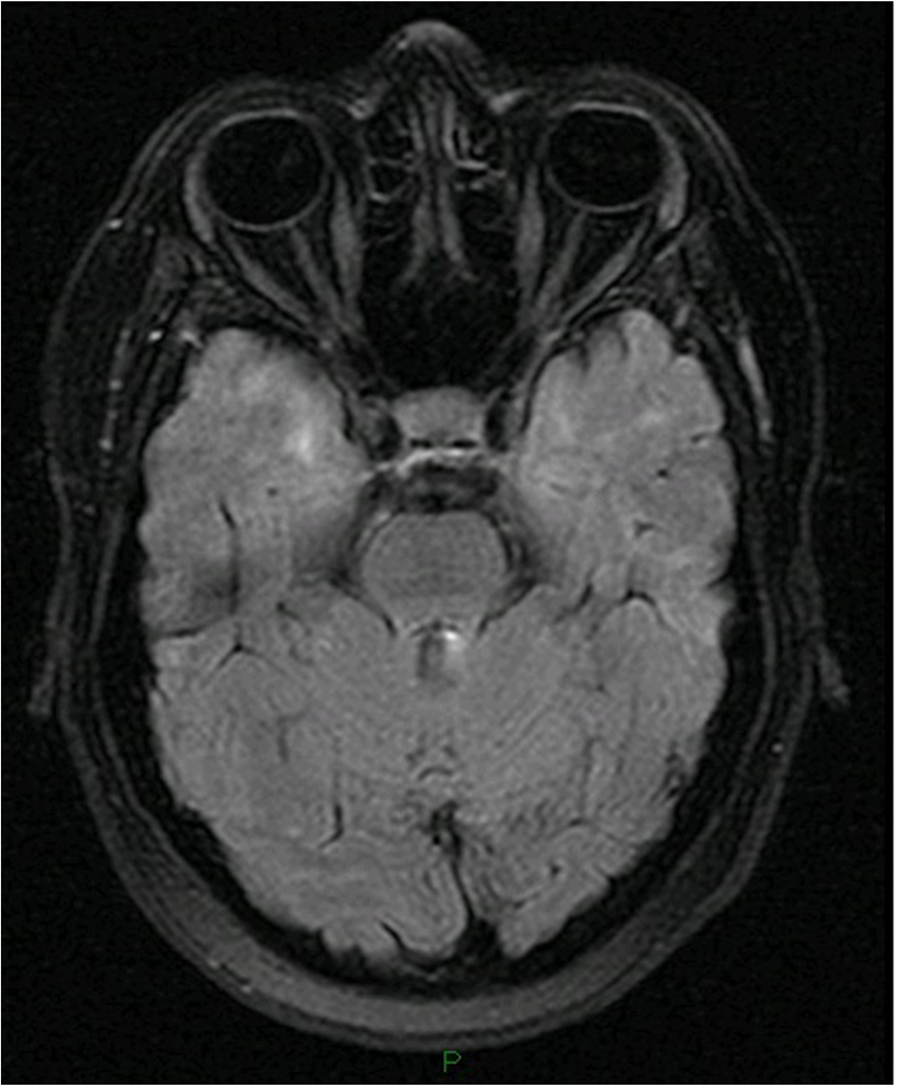
White matter lesions were also found in the temporal lobes.

Cranial computertomography showed numerous periventricular hypodense lesions without signs of acute cerebral infarction. Magnetic resonance imaging (MRI) was performed twice revealing general brain atrophy and multiple white matter lesions in all cerebral lobes, including the temporal region (Figures 1 and 2). No gadolinium enhancement and no abnormalities in diffusion weighted images (DWI) were found. No progression of the white matter lesions was detected from June to October on MRI scans.

Cardiologic examinations (including 24 h electrocardiogram and electrocardiography) and vascular ultrasonography showed no abnormalities. A lumbar puncture was performed to exclude inflammatory diseases of the central nervous system. In lab tests, serum creatine kinase (CK 194 U/l), C-reactive protein (CRP 16.4 mg/l), and γ-glutamyltransferase (GGT 63 U/l) were slightly elevated. Low-density lipoprotein cholesterol (LDL) levels were elevated (210.6 mg/dl) while high densitiy lipoprotein (HDL) was low (24.0 mg/dl), and the patient was given simvastatin. HbA1c was 7.00%. There weren’t any other cardiovascular risk factors.

**Figure 3:**
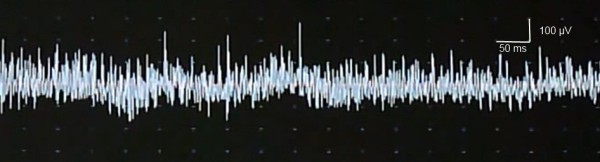
**Electromyography (EMG) of the anterior tibial muscle at rest.** The figure shows a myotonic discharge.

Electroneurography revealed a reduced nerve conduction velocity in the left sural nerve (35 m/s). In addition, we found a nerve block in the left peroneal nerve (fibula head). Somatosensory potentials of the right median nerve had a reduced amplitude (N20-P25 amplitude right 1.04 μV, left 3.23 μV) indicating an axonal damage. Electroneurography of the median nerve did not reveal any signs of carpal tunnel syndrome. P100-latencies of visually evoked potentials were delayed on both sides (P100 right 115 ms, left 111 ms). Surprisingly, electromyography of the anterior tibial muscle at rest showed many myotonic discharges (Figure 3).

On the basis of electromyography, genetic examination focusing on DM1 was initiated. There was a CTG-expansion of 200 ± 10 trinucleotides on one allele of the DMPK gene confirming diagnosis of DM1. The disease had been previously unknown to the family.

## Conclusion

This is the first case report of DM1 presenting as stroke-like episodes. While strokes may occur in DM1 patients due to cardiac involvement, MRI did not show any signs of acute cerebral infarction in this particular case. There was an amplitude reduction in somatosensory evoked potentials of the right median nerve (on the side of numbness) indicating axonal cerebral damage. The disease had been previously unknown to the family and the patient had no typical DM1 symptoms like limb weakness or myotonia. Physicians should be aware that extensive white matter lesions and stroke-like episodes may be the only symptoms of myotonic dystrophy. However, the observation does not necessarily constitute a causal relation.

## Consent

Written informed consent was obtained from the patient for publication of this case report and accompanying images. A copy of the written consent is available for review by the Editor-in-Chief of this journal.

## Competing interests

The authors declare that they have no competing interests.

## Authors’ contributions

All authors were involved in diagnostics, interpretation of the patient data, and writing of the paper. JR performed the electromyography and found myotonic discharges leading to genetic testing for DM1. All authors read and approved the final manuscript.
